# Why does circadian timing of administration matter for immune checkpoint inhibitors’ efficacy?

**DOI:** 10.1038/s41416-024-02704-9

**Published:** 2024-06-04

**Authors:** Abdoulaye Karaboué, Pasquale F. Innominato, Nicholas I. Wreglesworth, Boris Duchemann, René Adam, Francis A. Lévi

**Affiliations:** 1 UPR “Chronotherapy, Cancer and Transplantation”, Medical School, Paris-Saclay University, 94800 Villejuif, France; 2 Medical Oncology Unit, GHT Paris Grand Nord-Est, Le Raincy-Montfermeil, 93770 Montfermeil, France; 3 North Wales Cancer Centre, Ysbyty Gwynedd, Betsi Cadwaladr University Health Board, Bangor, LL57 2PW UK; 4 Cancer Chronotherapy Team, Division of Biomedical Sciences, Medical School, Warwick University, Coventry, CV4 7AL UK; 5 School of Medical Sciences, Bangor University, Bangor, LL57 2PW UK; 6 Thoracic and Medical Oncology Unit, Avicenne Hospital, Assistance Publique-Hôpitaux de Paris, 93000 Bobigny, France; 7 Hepato-Biliary Center, Paul Brousse Hospital, Assistance Publique-Hopitaux de Paris, 94800 Villejuif, France; 8 Gastro-intestinal and Medical Oncology Service, Paul Brousse Hospital, 94800 Villejuif, France; 9 Department of Statistics, University of Warwick, Coventry, UK

**Keywords:** Cancer immunotherapy, Lung cancer, Skin cancer, Renal cancer, Oesophageal cancer

## Abstract

**Background:**

Tolerability and antitumour efficacy of chemotherapy and radiation therapy can vary largely according to their time of administration along the 24-h time scale, due to the moderation of their molecular and cellular mechanisms by circadian rhythms. Recent clinical data have highlighted a striking role of dosing time for cancer immunotherapy, thus calling for a critical evaluation.

**Methods:**

Here, we review the clinical data and we analyse the mechanisms through which circadian rhythms can influence outcomes on ICI therapies. We examine how circadian rhythm disorders can affect tumour immune microenvironment, as a main mechanism linking the circadian clock to the 24-h cycles in ICIs antitumour efficacy.

**Results:**

Real-life data from 18 retrospective studies have revealed that early time-of-day (ToD) infusion of immune checkpoint inhibitors (ICIs) could enhance progression-free and/or overall survival up to fourfold compared to late ToD dosing. The studies involved a total of 3250 patients with metastatic melanoma, lung, kidney, bladder, oesophageal, stomach or liver cancer from 9 countries. Such large and consistent differences in ToD effects on outcomes could only result from a previously ignored robust chronobiological mechanism. The circadian timing system coordinates cellular, tissue and whole-body physiology along the 24-h timescale. Circadian rhythms are generated at the cellular level by a molecular clock system that involves 15 specific clock genes. The disruption of circadian rhythms can trigger or accelerate carcinogenesis, and contribute to cancer treatment failure, possibly through tumour immune evasion resulting from immunosuppressive tumour microenvironment.

**Conclusions and perspective:**

Such emerging understanding of circadian rhythms regulation of antitumour immunity now calls for randomised clinical trials of ICIs timing to establish recommendations for personalised chrono-immunotherapies with current and forthcoming drugs.

## Introduction

Cancer immunotherapy has developed with ups and downs since the late 1800s [1], with clinical trials mainly starting in the 1970s, and involving first live or inactivated microorganisms, then polyclonal or monoclonal antibodies, interferons, and interleukins. A leap forward in efficacy was achieved with the demonstration of unprecedented antitumor efficacy using immune-checkpoint inhibitors (ICIs) in chemo-refractory tumors [2]. ICIs are humanized monoclonal antibodies that target inhibitory receptors programmed cell death protein 1 (PD-1), cytotoxic T-lymphocyte antigen 4 (CTLA4) and programmed death-ligand 1 (PD-L1). PD-1 receptor is mainly expressed in activated T-cells, B-cells, natural killer cells, monocytes, and mesenchymal stem cells [3]. PD-L1 is expressed by many types of tumor cells and also constitutively expressed on dendritic cells, macrophages, mesenchymal stem cells, and bone marrow-derived mast cells [4, 5]. CTLA-4 (CD152) is predominantly expressed by T cells [6].

PD-1 is targeted by nivolumab, pembrolizumab, dostarlimab, sintilimab and cemiplimab, CTLA4 by ipilimumab and tremelimumab, and PD-L1 by atezolizumab, durvalumab, and avelumab. Receptor blockade by the specific antibody elicits an anti-tumor response, a process mostly orchestrated by cytotoxic T-cells [7, 8]. Specifically, antigen-presenting cells, e.g., dendritic cells, capture tumor antigens in the primary tumor tissues, then migrate to the tumor-draining lymph nodes, where T-cells are subsequently primed. Ultimately, primed T-cells infiltrate into the primary tumor sites and mediate tumor eradication.

These scientific advances led to the award of the 2018 Nobel Prize in Physiology or Medicine to Tasuku Honjo and James Allison for their discoveries in cancer immunology. Professor Honjo was awarded due to his discovery of the programmed cell death molecule-1 (PD-1) on T cells. Professor Allison discovered another important immunosuppressive molecule, i.e the cytotoxic T-lymphocyte antigen-4 (CTLA-4) [9]. However, clinical evidence has demonstrated heterogeneous responses in patients receiving ICIs, and primary or secondary therapy resistance is common [10]. Such between-patients differences in ICIs efficacy could result from chronopharmacologic effects, that would make immune cells and cancer cells respond differently to immunotherapy as a function of its administration timing along the 24 h time scale. Here, we first summarize the main results from fourteen retrospective studies, which have reported large and consistent differences in efficacy outcomes as a function of the Time of Day (ToD) of ICI infusions over the past 2 years. We then outline the physiologic and molecular mechanisms at work within the circadian timing system (CTS), which rhythmically regulates immune cell functions and trafficking, besides cellular metabolism and proliferation over the 24-h. Thus, circadian time dependencies characterize both the tolerability and efficacy of chemotherapeutic and immunotherapeutic agents, thus supporting their optimization according to circadian rhythms, so-called chronotherapy [11, 12], as well as its personalization based upon circadian biomarkers metrics.

## Improved ICI’s efficacy through early time of day infusions

**Fig. 1:**
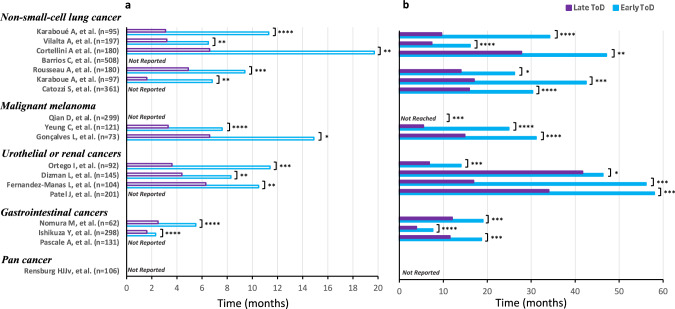
Bar graph of efficacy endpoints of immune checkpoint inhibitors in compared timing groups from 18 studies involving a total of 3250 patients with different cancer types. **a** Median progression-free survival data. **b** Median overall survival data. Violet bars correspond to late time-of-day ICI dosing. Blue bars correspond to early time-of-day of ICI administration. The main characteristics of these studies are summarized in Table S1. Note: Qian et al. study results not shown despite statistically significant differences in overall survival in favor of the early time-of-day group, because median values were not reached Reported *p* values from timing groups comparisons of PFS or survival curves: *P > 0.20; ** 0.05 < *p* ≤ 0.20; *** 0.01 < *p* ≤ 0.05; ****0.001 < *p* ≤ 0.01.

In seminal clinical observations, one of us (AK) first noticed a strikingly longer follow-up for those patients who received most nivolumab infusions in the morning as compared to those treated mostly in the afternoon for metastatic non-small cell lung cancer (mNSCLC) [13]. The results suggested a possible role for the CTS on immunotherapy effects, similar to that found for chronomodulated chemotherapy [14–16]. Seventeen of eighteen retrospective studies have now reported prolonged progression-free and/or OS through early ToD of ICI infusions in five different cancer types (Fig. 1, Table S1).

### ToD of immunotherapy for metastatic non-small-cell lung cancer patients

In 95 mNSCLC patients on second-line nivolumab, both PFS and OS were 3 to 4 times as large in those receiving the majority of nivolumab infusions before 12:54, i.e. the median value of the per-patient median timing of infusions, with respective Hazard Ratios of an earlier progression or an earlier death of 0.258 [95% CL, 0.115–0.580] and 0.174 [0.082–0.370] [11]. Predominantly morning administration of nivolumab was significantly more effective irrespective of performance status and tumor PD-L1 expression, as shown in multivariable analyses (PFS, *p* = 0.001; OS, *p* < 0.001). The larger the proportion of infusions given before 12:54, the better the PFS and OS outcomes. Thus, median OS was 34.2 months for those patients receiving at least 67% of ICIS infusion before 12:54, 12.4 months for those given at least 67% of ICI infusions after 12:54 and 15.3 months for those receiving at least 33% of ICIs before and 33% after 12:54 (*p* = 0.023).

Two studies investigated whether ToD of infusions influenced the efficacy of single-agent pembrolizumab as 1st or 2nd line in 180 mNSCLC patients each [17, 18]. The early ToD group comprised patients who received <20% infusions after 16:30 whilst the late ToD group involved patients who received >20% infusions after 16:30, according to the initial methodology proposed by Qian et al. [12]. As compared to the late ToD group, the early ToD groups displayed longer PFS in both studies, i.e. 19.7 vs 6.6 months, Log Rank, *p* = 0.056 [17], and 9.4 vs 4.9 months, *p* = 0.020 [18]. Median OS was also largely prolonged in the early vs late ToD group, yet not significantly so (Fig. 1). The relevance of the ToD of the initial 4 ICI infusions was shown in a study involving 197 mNSCLC [19], using 12:00 noon as a time cut-off. Their early ToD group consisted of patients receiving at least one of the initial 4 ICI infusions before noon, whilst their late ToD group involved those receiving all initial four infusions after 12:00 noon. Median PFS was 6.5 months in the early ToD group and 3.2 months in the late group (*p* = 0.066). Median OS were 16.1 and 7.4 months, respectively (*p* = 0.003). In a multivariate analysis, HR was 0.42 [0.27–0.63] (*p* < 0.001) for PFS and 0.54 [0.35–0.84] (*p* = 0.007) for OS.

The critical relevance of early ToD of infusions over the initial 4 treatment courses was confirmed in 97 mNSCLC patients receiving 1st line pembrolizumab as a single agent (*N* = 41) or combined with chemotherapy (*N* = 56) [20]. Thus OS was significantly prolonged through the administration of three or four of the 4 initial pembrolizumab infusions before 11:45 (early ToD group) as compared to one or two (late ToD). Thus, respective 2-year survival rates were 65% and 38% (*p* = 0.010). Multivariable analysis confirmed that the delivery of a majority of the four initial pembrolizumab-based courses in the morning was an independent predictor of a longer OS (HR = 0.28 [0.13–0.64], *p* = 0.003).

In a cohort of 361 patients with metastatic NSCLC, Catozzi et al. also found that early ToD infusions of pembrolizumab, nivolumab, atezolizumab, durvalumab, or avelumab significantly prolonged overall survival. They identified 11:37 as the best discriminant cut-off time. As a result, median overall survival times were 30.3 months for the patients whose majority of ICI infusions occurred before 11:37as compared to 15.9 months for those treated later in the day (*p* = 0.0024) [21].

All the above studies also showed that the early ToD groups received significantly more ICI infusions as compared to the late ToD groups since efficacy was best on early ToD treatment. Such an explanation is consistent with all the results where an ICI-based treatment arm proved more effective in randomized trials. Thus ICIs withdrawal is mostly related to disease progression [22]. As a result, time to treatment failure was also found to be longer following early vs late ToD administration of nivolumab, pembrolizumab or atezolizumab in 129 mNSCLC patients, with respective median times of 14 months (CI 95%, 8.9-23.4) vs 4.9 months (2.8-13.5) [22]. There were not enough events for any comparative survival assessment at the time of the report.

### ToD of immunotherapy for metastatic malignant melanoma patients

In 299 patients with stage IV malignant melanoma in the MEMOIR Cohort Study, Qian et al. [12] first reported that more frequent early ToD dosing of nivolumab, pembrolizumab and/or ipilimumab nearly doubled OS as compared with late ToD administrations. The early or late ToD groups involved the patients who had received less than 20% of ICIs infusions before 16:30 or 20% or more infusions after 16:30, respectively. In a propensity score-matched (PSM) analysis of 146 patients, the relative risks of earlier disease progression or death were nearly twice as low in the early vs late ToD group. Median OS was 4·8 years [3·9–not estimable] for the late ToD group, and was not reached for the early ToD group (HR = 2·04 [1·04–4·00]; *p* = 0·038). ICI timing was an independent prognostic factor for OS in multivariable analyses and remained unaltered using different types of ICIs, or prior corticosteroids or brain radiotherapy.

The above ToD effects were confirmed in a cohort of 121 patients with advanced melanoma [23]. The patients receiving all initial four infusions in the afternoon (after 12:00– noon) displayed worse PFS (3.3 vs 7.6 months; *p* = 0.009) and worse OS (5.5 vs 24.9 months; *p* < 0.001) compared to those given at least one of the initial four infusions in the morning. The results were confirmed using multivariable analyses.

Another retrospective study involving 73 patients with stage IV melanoma further showed that those patients receiving more than 75% of infusions of nivolumab, pembrolizumab, or nivolumab + ipilimumab in the afternoon had a shorter median survival as compared with those receiving more morning infusions (14.9 vs. 38.1 months; HR = 0.45 [0.23–0.86]; *p* < 0.01) [24].

### ToD of immunotherapy for metastatic urothelial or renal cancers

A significant association between early ToD of ICI infusions and improved patient outcomes has been reported for metastatic urothelial or renal cancers in four studies.

In 92 patients receiving single-agent ICIs for metastatic urothelial cancer in Spain or Italy, Ortega et al. [25] showed that those receiving fewer than 20% ICI infusions before 16:30, had longer PFS and OS, compared to those receiving 20% or more ICI infusions after 16:30. Median PFS ranged from 11.4 to 3.6 months (HR = 2.66 [1.53–4.63] (*p* = 0.001) and median OS varying from 14.0 to 6.8 months (HR = 2.62 [1.48–4.63] (*p* = 0.001). Early ToD of infusions also improved tumor response rate (59.3% vs 16.0%). In 201 patients receiving pembrolizumab (8% of the patients), nivolumab (61%), or dual nivolumab/ipilimumab (31%) for stage IV renal cell carcinoma (RCC), Patel et al. [26] observed that the patients receiving 25% or more of their ICI infusions in the morning (before 13:00) had significantly longer OS compared to those receiving more than 25% of infusions after 13:00. Median OS were 58 and 34 months, respectively (HR = 0.51, [0.29–0.89] (*p* = 0.017). Early ToD was prognostic of longer survival, independently of age, sex, tumor histology, liver or brain metastases, pre-treatment LDH, and initial ICI. Similar results were found in a study involving 56 patients receiving anti-PD-1/PDL-1 as 1st or 2nd line treatment for metastatic RCC [27]. Those patients receiving 20% or more ICI infusions after 16:30 experienced worse OS (HR = 3.1 [1.27–7.36] (*p* = 0.01), shorter time on treatment (HR = 2.5 [1.21–4.99] (*p* = 0.013) and shorter time to next treatment line (HR = 1.9, [0.96–3.71] (*p* = 0.067) as compared to those who received less than 20% of the infusions after 16:30. In 145 patients treated with nivolumab alone, or in combination with ipilimumab as first- or second-line treatment for metastatic RCC, Dizman et al. [28] showed consistent trends towards improved response rate, prolonged time-to-treatment failure, and OS among those patients receiving less than 25% of ICI infusions after 16:30.

### ToD of immunotherapy for metastatic gastrointestinal cancers

In 62 patients treated with nivolumab for recurrent or metastatic squamous cell carcinoma of the esophagus, significantly superior response rate, PFS, and OS resulted from the administration of the first infusion of nivolumab before 13:00 compared to its administration after 13:00 [29]. Response rate and PFS were also significantly larger in those patients who received the majority of infusions before 13:00 during the first 3 months of treatment. In contrast, no significant differences were found in response rate, PFS, and OS as a function of whether the majority of all nivolumab infusions was given before or after 13:00.

In 248 patients receiving nivolumab monotherapy for metastatic gastric cancer, Ishizuka et al. observed significant difference in response rate, PFS and OS according to whether patients received more or fewer than 70% of ICI infusions before 14:00. The respective efficacy data were: ORR, 16.9% vs 3.3%, (*P* = 0.01); PFS, 2.3 vs 1.6 months (HR = 0.65; *P* < 0.01); OS, 7.6 vs 3.9 months, (HR = 0.64; *P* < 0.01) [30].

In 131 patients receiving atezolizumab ± bevacizumab as first-line treatment for advanced hepatocellular carcinoma, Pascale et al. showed that the administration of the first two treatment courses after 13:00 was associated with a significant reduction of median OS as compared to the administration of at least one of the two first courses before 13:00 (11.5 months vs 18.7 months), *p* = 0.015). This ToD-related difference in OS outcome was supported by multivariate analysis [31].

A meta-analysis evaluated the consistency of ToD-related efficacy findings, using published data from 13 studies involving 1663 patients with advanced non-small-cell lung cancer (47%), renal cell carcinoma (24%), melanoma (20%), urothelial cancer (5%), or esophageal carcinoma (4%). The patients had received anti-programmed cell death protein 1, or anti-programmed death-ligand 1 (98%) and/or anti-cytotoxic T-lymphocyte- associated protein 4 (anti-CTLA-4) (18%). The early ToD groups had nearly twice as long OS and PFS, as compared to late ToD-treated patients. Respective overall HRs were 0.50 [95% CI, 0.42–0.58]; *P* < 0.00001) for OS, and 0.51 [0.42–0.61]; *P* < 0.00001) for PFS [32].

A single study involving 106 patients with more than six different types of solid tumours found no difference in PFS or OS between early and late ToD infusion of pembrolizumab. No role was found for cut-off points at 12:00 noon or at 15:06 (median) or at 16:30, nor for the proportion of cycles given after this cut-off point (20% or 50%). The authors ascribed this negative result to the large heterogeneity of their patient population [33].

Thus, 17 of the 18 above study reports, involving a total of 3144 metastatic cancer patients from nine countries in 4 continents, have consistently indicated improved patient outcomes following predominant early ToD administrations of ICIs (Fig. 1a, b). However, the definition of the threshold for early ToD varies from 11:37 to 16:30, whilst the definition of the proportion of late ToD infusions associated with poor ICIs efficacy ranges from 20% to 75%.. Four studies further highlighted the critical relevance of early ToD over the initial 2-months of ICI treatment. Thus, there is a clear need both for improving precision regarding the determination of optimal timing and for assessing the current evidence for the circadian mechanisms at work for cancer chronotherapy.

## From circadian rhythms to cancer chronotherapy

### The circadian timing system

**Fig. 2:**
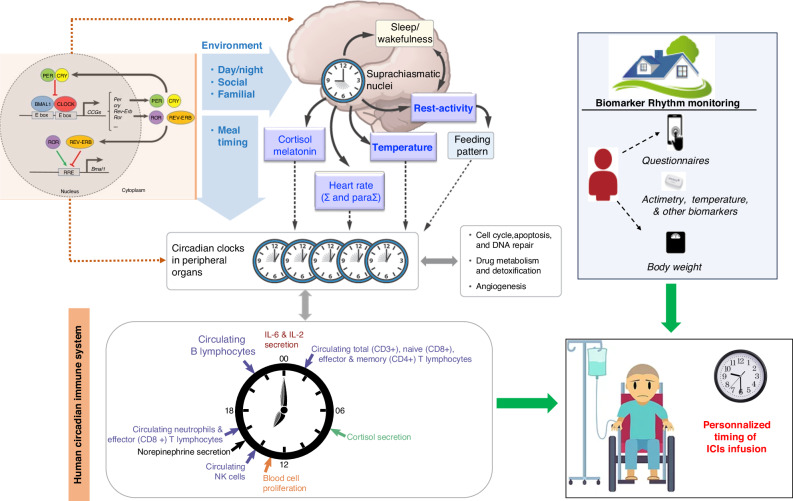
Interactions between the circadian timing and the immune systems. Ʃ and paraƩ = sympathetic and parasympathetic systems.

Circadian rhythms consist of endogenous biological oscillations with a period of about 24 h, which characterize most physiological parameters, thus contributing to well-being and health [34–40]. In mammals, circadian rhythms regulate sleep-wakefulness, rest-activity, appetite, muscular and cognitive performance, body temperature, hormonal secretions, cellular metabolism, proliferation, and death, as well as immune cells production, functions and trafficking [41]. Circadian rhythms display precise 24-h periods, following their synchronization with 24-h environmental cycles, and especially the regular alternation of light and darkness over the 24 h [38, 42]. Other environmental cues that influence circadian rhythms include timed physical activity and rest, socio-professional and familial interactions, and feeding patterns [43–46]. The circadian coordination and adjustment to environmental cues mostly operate through the suprachiasmatic nuclei (SCN), a hypothalamic pacemaker located above the optic chiasm [47]. Most circadian rhythms are suppressed following SCN physical ablation or functional alteration, through chronic jet lag for instance [48–51]. SCN-dependent rhythmic signals coordinate genetic molecular clocks that reside within each mammalian cell, resulting in a hierarchical clocks network that constitutes the circadian timing system (CTS) [38, 52]. Indeed, mammalian cells are endowed with a molecular clock involving 15 clock genes (NPAS2, PER2, DBP, ARNTL, PER3, NR1D1, CRY1, NR1D2, CLOCK, CRY2, BHLHE40, PER1, BHLHE41, RORA, and TIMELESS), that interact with each other through three transcription/post-transcription regulatory loops, thus generating circadian oscillations in individual mammalian cells. The BMAL1::CLOCK (or NPAS2) protein dimers, in particular, play a key role in the molecular clock through the activation of the transcription of clock genes *Per’s*, *Cry’s and Rev-erb’s* [44, 53] (Fig. 2). Dynamic single-cell reporter studies have revealed the tight coupling between the molecular circadian clock and the cell cycle, in individual non-malignant cells, whilst such coupling might be altered in individual cancer cells [54]. In healthy mouse and/or human tissues, cell cycle events, DNA repair, apoptosis and autophagy are controlled by the circadian clock [55]. Experimental studies have further highlighted the essential roles of tissue-specific regulation and intercellular signaling in clock synchronization.

### Chronopharmacology and chronotherapy

Comprehensive reviews have summarized the evidence of circadian time-dependencies in drug effects, i.e. chronopharmacology, and the main mechanisms that determine chronopharmacokinetics and chronopharmacodynamics [38, 44, 56–59]. Drug exposure is moderated by the circadian regulation of Absorption, Distribution, Metabolism, Elimination and Toxicities (ADMET) mechanisms at molecular, tissue and organ levels. Circadian rhythms characterize most bioactivation, detoxification and elimination processes at the transcription, protein and enzymatic levels in the liver, the chief drug-metabolizing organ, as well as in the intestine, kidney, lung, etc [60]. Recently, sex dimorphism has emerged for circadian drug responses and their molecular mechanisms, thus supporting sex-specificities in optimal circadian-based treatments, i.e. chronotherapy [61, 62].

### Circadian rhythms in cancer biology

The occurrence of circadian patterns in clock gene expressions has been shown for several but not all rodent or human cancer cell lines in synchronized cell culture studies [63–66]. Large between-tumor variations characterize both the extent of clock gene expressions and their circadian patterns in cancer tissues from rodents or humans. A metric for molecular circadian clock functionality and timing has recently been developed using Time Teller, an artificial intelligence algorithm, that proved able to model the molecular clock from a single tissue biopsy transcriptome or RNAseq [67]. Time Teller application to human oral mucosa and breast cancer omics data reveal a molecular clock disruption in nearly half of the patients’ tumors. TimeTeller has further revealed that the survival of 209 patients with primary breast cancer treated with neoadjuvant chemotherapy was significantly less when circadian molecular clocks in cancer tissue were functional, probably due to the reduced effectiveness of chemotherapy on cancer cells with functional clocks [67]. Interestingly, the expressions of common oncogenic drivers or inhibitors such as RAS, c-MYC, NOTCH and TP53 are rhythmically controlled by the circadian clock, and also interact with it, thus supporting an overarching regulatory role of the circadian clock in cancer processes and precision cancer treatments.

Other algorithms that estimate molecular circadian clock functionality and timing have been developed such as TimeSignature [68], ZeitZeiger [69], and CYCLOPS [70]. However, the key point differentiating TimeTeller from the other algorithms is that, apart from identifying timing deviations, these do not provide any other assessments of clock functionality or other quality controls on the individual timing assessments [67].

### Cancer chronotherapy

Cancer chronotherapy aims at the delivery of anticancer agents at a time (on the 24-h scale) when efficacy can be maximized and/or toxicity can be minimized. As a result, the circadian dosing time can improve or increase the extent of treatment toxicities by up to fivefold, as shown for more than 50 agents in experimental models, and 12 of them in cancer patients [62]. Most importantly, the dosing time associated with least toxicity has achieved similar or improved efficacy both in rodents and in patients [71, 72]. Clinical trials have been conducted from the evidence provided by experimental studies in nocturnal rodents, and more recently in synchronized cell cultures as well [38, 56, 65]. Overall, the clinical relevance of daily timing has been investigated for anticancer agents in human colorectal, lung, breast, pancreas, kidney, bladder, endometrium, ovary, head and neck or hematologic cancers [62]. The discovery of oxaliplatin efficacy in colorectal cancers resulted from its chronopharmacologic administration, from Phase I to large international Phase III trials [73–75]. An individual patient-level meta-analysis involving data from 345 females and 497 males with metastatic colorectal cancer revealed that males lived significantly longer on chronomodulated oxaliplatin-5-fluouracil-leucovorin rather than on conventional chemotherapy, whilst the opposite was observed for women [76]. Further experimental and clinical data support an about 6-h delay in the optimal timing of oxaliplatin, 5-fluorouracil and irinotecan, both in mice and in cancer patients [62]. Altogether, a recent systematic review involving 18 randomized, controlled studies including a total of 2547 patients concluded that most studies provided evidence of the reduction of toxicity resulting from chronomodulated chemotherapy, while efficacy was maintained or improved [77].

## Mechanisms at work for cancer chronoimmunotherapy

### The circadian immune system

Oscillating molecular circadian clocks have been identified in all the cells that participate in immune system functions [78–80]. Immune cells express circadian clock genes and display 24-h mRNA and/or protein rhythms for a wide array of functional genes. As a result, circadian rhythms characterize (i) the synthesis and release of cytokines, chemokines and cytolytic factors, (ii) the daily gating of the immune response occurring through pattern recognition receptors, (iii) phagocytosis, (iv) migration to inflamed or infected tissue and immune cells trafficking, (v) cytolytic activity, and (vi) proliferative response to antigens [81]. Consequently, alterations of circadian rhythms can lead to profound disturbances in immune responses.

In experimental models, the encounter of an immune challenge given during the middle of the light span, i.e. at Zeitgeber 7 (ZT7) 7 h after light onset, when mice are resting, has been associated with an enhancement of immune responses compared to exposure in the middle of the night (ZT19) when mice are most active [41, 82, 83]. Thus, an immune stimulus caused more dendritic cells to migrate faster from the skin to the lymph nodes following its application at daytime, as compared to night-time. The number of T-cells in lymph nodes was higher during the day, and T-cell movement from blood to lymph nodes was controlled by the circadian expression of the homing molecule Intercellular Adhesion Molecule 1 (ICAM-1) [82]. Mathematical modeling revealed that the presence of dendritic cells and T-cells in the lymph node at daytime, during the rest span of mice, increased the probability of immune interactions between antigen-presenting dendritic cells and antigen-recognizing T-cells [78]. Circadian rhythms also moderated the proliferation of T-cells in the lymph nodes at a later stage of an adaptive immune response. One week after an immune stimulus consisting of OVA peptide-loaded dendritic cells, T-cells proliferated more at daytime compared to nighttime, with largest daytime expression of proteins involved in immune regulation [84]. These rhythmic patterns were suppressed in mice lacking clock gene Bmal1 expression in T-cells [85] or in those with constitutive CLOCK gene mutation [84].

The timing when the immune challenge was given also affected the later stages of the adaptative response. The B-cells from mice vaccinated in the daytime produced more antibodies at 14 days and more antigen-specific antibodies at 28 days, compared to mice vaccinated at night [86, 87]. Day-vaccinated mice also had a stronger virus-specific T-cell response at 28 days. This effect was not seen in mice lacking Bmal1 in T-cells [86, 87].

**Fig. 3:**
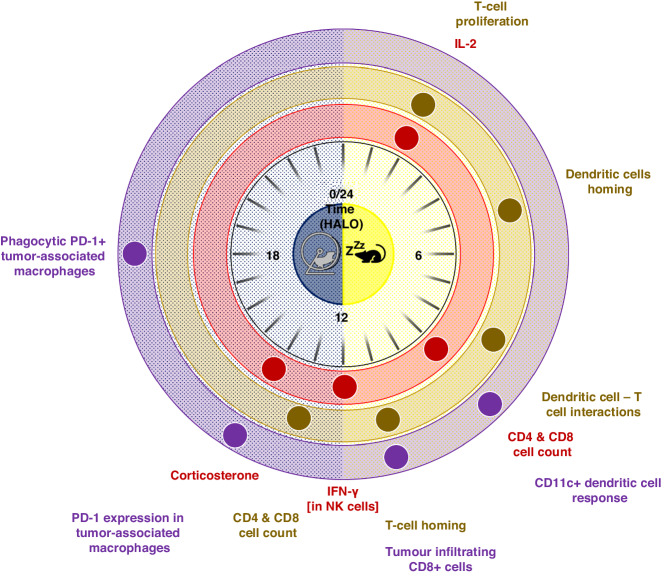
Schematic polar representation of the distribution of peak times of selected rhythmic immune functions involved in antitumor immunity in rodent models over 24 h. Peak time data are shown in three key compartments, i.e. bloodstream [red], lymph nodes [green], and tumor immune microenvironment [purple]). The circle in the center depicts the light-dark synchronization schedule of nocturnally active mice or rats. HALO Hours After Light Onset.

Experimental results combined with mathematical modeling, revealed that the oscillating nature of one stage of the immune response, such as T-cell trafficking, feeds into and is necessary for the next stage such as T-cell proliferation, which is also rhythmic. This mechanism enables the immune response to remain rhythmic for several weeks after an initial challenge [87]. Figure 3 provides an overview of the peak times of relevant cellular, humoral and molecular immune parameters in the circulating blood, the draining lymph nodes and the tumour immune microenvironment in nocturnal rodent models [87–91].

In healthy humans, circadian variations with large amplitudes characterize the circulating counts of most blood cells, including total lymphocytes and their subsets. Total lymphocytes and mononuclear cell counts reach a low point in the morning hours, i.e. between 08:00 and 10:00 then gradually increase with a daily maximum between midnight and 02:00 at night [92]. All subpopulations of interest of T(CD4^+^) and T(CD8^+^) cells including naive, central memory, effector memory, and effector T cells display distinct and significant circadian rhythms in absolute cell counts, except for effector T(CD4) cells. Most lymphocyte subset counts nearly double over the 24-h time scale. Acrophases also clump in the early night span, thus ranging from 01:31 to 02:41 for naive, central memory, and effector memory T(CD4) and T(CD8) cells. In contrast, circulating counts in both effector T(CD8) cells and natural killer cells peak in the morning or early afternoon hours [83]. Immune cell trafficking and functions are regulated in part by hormonal secretions. For instance, cortisol [93] is rhythmically secreted by the adrenal cortex in the early morning hours with reduced levels at night. Cortisol, increases the expression of CXCR4 to mediate bone marrow homing with a 3-h delay, thereby negatively regulating the counts in naive and memory T-cells [83]. Epinephrine is secreted by the adrenal medulla primarily in the afternoon hours [94]. It recruits natural killer cells and effector T cells from the marginal pool to the circulation by increasing the expression of β-adrenergic receptors and the chemokine receptor CX3CR1 and reducing the expression of adhesion molecules [83]. Adrenergic signaling also regulates the expression of vascular cell adhesion molecule-1 [95] in the bone marrow microenvironment to induce homing of leucocytes to the bone marrow and in muscles late in the activity phase [95]. Hence, trafficking to peripheral sites occurs simultaneously, thereby resulting in anti-phasic abundance in the bloodstream.

**Fig. 4:**
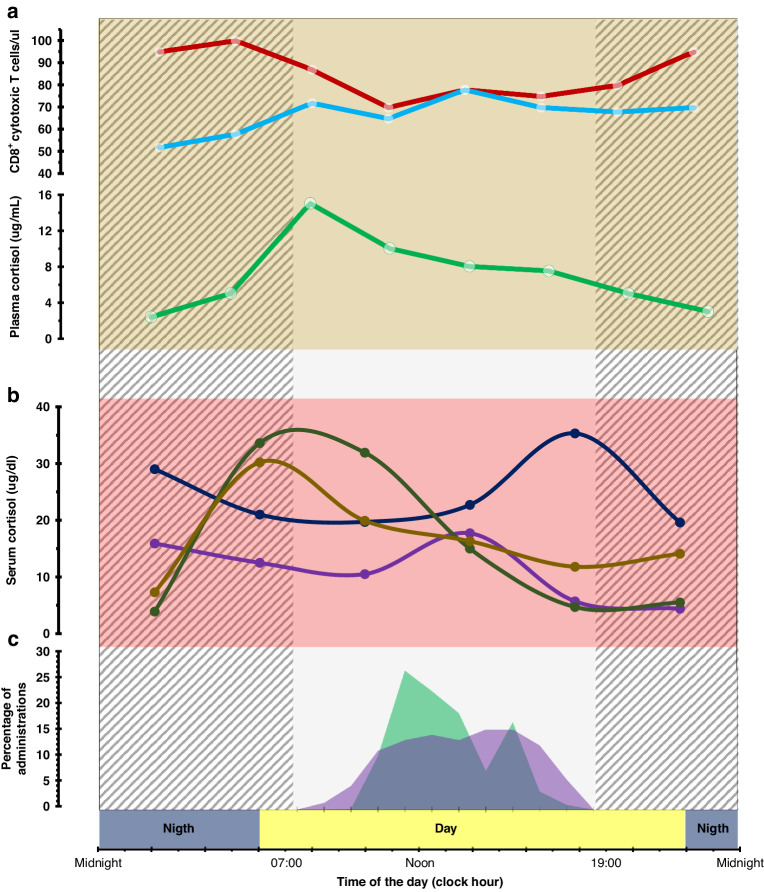
Spectrum in peak times of selected human circulating immunological factors with a proven 24-h rhythm relevant to cancer immunotherapy. **a** Healthy (male) subjects. CD8 cytotoxic Central memory [red], effector [blue], plasma cortisol [green]. **b** Salivary cortisol in four individual patients with advanced cancer. **c** Distribution of daily timings of ICI administration in two studies: Karaboué et al. Cancers [11] [green] and Qian et al. Lancet Oncol. [12] [purple].

Circadian rhythms regulate macrophage-mediated immune responses through time-of-day-dependent regulation of macrophage function [96]. Circadian disruption induced the loss or inversion of daily patterns of M1 (proinflammatory) and M2 (anti-inflammatory) macrophages in the spleen [97]. The immune time-of-day variation has also been shown in the magnitude of the response to vaccination. Thus, preclinical and clinical studies have shown that a weaker adaptive immune response is induced when vaccinations are given in the evening than when given earlier in the day [98–102]. Figure 4 critically summarises a snapshot of pertinent available data on the 24-h rhythms in immune functions in peripheral blood demonstrated in humans using repeated measures. These results support strategies for optimizing therapeutic options through treatment timing of administration in a personalized and precise fashion.

### Experimental cancer chronoimmunopharmacotherapy

Improved treatment responses have resulted from circadian-timed delivery of interleukin-2 and interferons in laboratory rodents. In B16 melanoma-bearing mice, maximum antitumor activity was achieved following dosing at ZT4, during the early stage of the diurnal rest span of mice, for recombinant human interferon (IFN)α A/D and at ZT16 for recombinant murine IFN-γ, i.e. shortly before the middle of the nocturnal activity span [103]. The mRNA expression of IFN receptor was rhythmic in tumor cells, and peaked synchronously with the proportion of S-phase cells, thus supporting a link between the cell cycle and immunologic tumor responses potential. Highest interferon receptor expression occurred near the middle of the rest phase of mice [104].

The administration of IFN-α or IFN-γ disrupted circadian gene Per2 mRNA expression, both in the SCN and in peripheral organs, as well as the circadian rhythm in rest-activity and body temperature, following daily dosing at ZT12 but not at ZT0. The study further revealed the ability of IFNs to disrupt both the central circadian pacemaker and peripheral clocks following dosing around the beginning of the activity span (ZT12) [104]. The circadian disruption potential of IFN was further shown through the continuous administration of IFN-α to mice using a subcutaneous implanted pump. Constant rate IFN-α infusion decreased the rhythm amplitude of locomotor activity, body temperature, leukocyte counts, and plasma corticosterone levels and suppressed the oscillation in the expression of clock genes in the liver compared to repeat daily administrations at ZT2 [105]. The chronopharmacology of interleukin-2 (IL-2) was shown in tumor-bearing rats, where constant rate infusions of IL-2 induced a 37.5% mortality rate and a 25% objective tumor response rate, whereas animals receiving a “day cycle” of IL-2 had no mortality and a 100% objective response rate [106]. Clinical Phase 1 and 2 studies have shown good tolerance despite a significant increase in dose intensity with a circadian infusion schedule compared to standard or flat continuous infusion schedules of recombinant alpha-interferon-2b in melanoma or renal cell cancer patients [107, 108]. IL-2 chronotherapy also proved to be safe, moderately toxic and active in metastatic RCC patients [109].

Circadian rhythms in key mechanisms of ICI’s efficacy were recently demonstrated in mice. Circadian *Pdcd-1* mRNA expression was found in tumor-associated macrophage (TAMs), whilst PD-1-positive TAMs count also displayed a significant circadian rhythm in B16/BL6 melanoma transplanted into C57BL/6J mice [88]. *Pdcd-1* was further characterized as a clock-controlled gene [88]. In this mouse model, BMS-1, a small molecule inhibitor of PD-1/PD-L1, was significantly more effective following its administration at ZT18, near the middle of the activity span, in comparison with ZT6. Both circadian times, respectively correspond to the rise and decrease in PD-1 expression on TAMs. Although a two-timepoints comparison cannot identify the optimal time of administration of BMS-1, the findings suggest that the circadian expression of PD-1 on TAMs, but not in circulating monocytes, could help select the most appropriate time of day to administer PD-1/PD-L1 inhibitors. Wang et al. [89] further demonstrated that the anti-tumor immune responses were ToD-dependent. Fourteen days after the subcutaneous engraftment of a fixed melanoma cells load, tumor volumes were significantly larger in those mice engrafted ZT21 (in the mid-to-late activity span of mice) as compared to ZT9 (in the mid-to-late rest span). This difference was abrogated in mice lacking both adaptive and innate immune cells. Interestingly, T (CD8) cells, the main target cells of ICIs were significantly more numerous in the tumors at ZT9 compared to ZT21. This difference was abrogated in mice whose Bmal1 expression was specifically suppressed in dendritic cells. Thus both above reports identify a 9-h long time span, ranging from ZT9 to ZT18, within which ICIs might be most effective. Further studies are yet mandatory to identify the optimal circadian times of ICIs, and the circadian biomarkers that will help the personalization of the chronoimmunotherapy schedules.

### Chronopharmacokinetics

In humans, ICIs have an elimination half-life that ranges between 14 and 27 days, and a mean clearance of about 0.36 L/day [18, 110, 111]. As a result, the steady state is usually reached after 2–4 months [112]. Additionally, it takes 32.4 weeks to reduce T-cell PD-1 occupancy by 50%, after nivolumab discontinuation [113]. These data reveal that ICIs timing might only moderate their pharmacokinetics over the initial 2–4 months of treatment, i.e. before the reach of the steady state of ICIs blood concentrations. Furthermore, the pharmacokinetics of monoclonal antibodies have peculiar aspects and are influenced by target-mediated drug disposition and drug clearance [114], which vary over time [115, 116]. Preclinical studies have shown that pembrolizumab biodistribution depends on PD-1-mediated uptake into lymphoid organs, which oscillates with a circadian rhythm. Thus, there might be circadian changes in the clearance of monoclonal antibodies from tissues with a crucial impact on T-cell antitumor function [117].

These observations call for careful investigations of ICI chronopharmacokinetics, as potential mechanisms for the dosing time dependencies in ICI efficacy over the initial treatment cycles.

## Circadian biomarkers towards personalized chronoimmunotherapy

The main physiological host biomarkers that inform CTS function and timing include the 24-h patterns in rest-activity, body temperature, and heart rate, as well as cortisol and melatonin secretions. These rhythms help coordinate peripheral clocks over the 24 h. They can be moderated by sex, age, as well as cancer type and stage [49, 118, 119]. In metastatic cancer patients, such circadian biomarkers either remain similar to those in healthy people, or be shifted, damped, or altogether suppressed. Predictable chronopharmacologic effects require well-coordinated and functional circadian clocks [38, 56]. Between-patient differences have been identified for numerous host circadian biomarkers [57, 120, 121]. Furthermore, robust, altered or suppressed clock gene expressions and circadian clock functionality have been found in the tumors from cancer patients [67, 122]. It can thus be hypothesized that the patients with rhythmic biomarkers could benefit the most from chrono-immunotherapy.

### Circadian patterns in rest-activity

In mammals, the circadian rhythm in rest activity and that in body temperature are generated by the SCN [123], thus representing biomarkers of the coordination activity of the central circadian pacemaker [123]. In humans, the dampening of the circadian amplitude in rest-activity rhythm, measured with wrist actimetry for a week, has been associated with a significant increase in both the incidence of cancer and other diseases and all causes of mortality among the 92614 participants in the UK Biobank study [124]. In cancer patients, the most clinically relevant metric of the rest-activity rhythm is the dichotomy index (I < O), a measure of the percentage of in-bed activity counts that are less than the median of out-of-bed counts. Nearly half of 436 patients with metastatic colorectal cancer patients had robust rest-activity rhythm, with an I < O of 97.5% or more, i.e. similar to healthy subjects [125], whilst 25% had damped rhythms, and a further 25% had severe circadian disruption with an I < O below 96% [126]. Low I < O values consistently predicted for both poor PFS and OS in a pooled analysis involving these 436 patients [126], and for OS in an independent meta-analysis of 6 studies in a total of 659 cancer patient [127]. Large between patients differences have been reported for the rest-activity circadian pattern in several studies Involving patients with advanced or metastatic lung or esophagus cancer or melanoma, i.e. populations similar to those where early ToD ICIs improved outcomes. The clinical relevance of rest-activity circadian disruption has also been shown especially in lung cancer patients [128–131]. Walking exercise improved sleep quality, as assessed with the Pittsburgh Sleep Index questionnaire, and the rest-activity dichotomy index I < O, only in the lower I < O group, while no effect of the intervention was found in the good I < O group or in the whole population [132]. The restoration of a near-normal rest-activity rhythm with gefitinib was associated with decreased symptom severity and improved quality of life in a pilot study involving 10 NSCLC patients [133]. In such patients, rest-activity rhythm disturbances have been frequently associated with alterations in cortisol and melatonin secretions, core body temperature, and/or circulating immune cell subset counts [134].

### Circadian patterns in cortisol or melatonin secretions

Disrupted circadian patterns have been identified for cortisol secretion in individual patients with advanced or metastatic breast or ovarian cancer [135]. Such disruption was further shown as an independent prognostic factor of survival in metastatic breast cancer patients [136], as well as for advanced or metastatic patients with NSCLC [137] or RCC [138], but not for those with metastatic colorectal cancer [139].

In NSCLC patients, Sephton et al. [137] observed a significant increase in early mortality among the 29 patients with flattened diurnal salivary cortisol slope compared with the 32 patients with steep slopes that suggested a near normal circadian pattern. She found that disrupted circadian cortisol profiles were associated with both low circulating total and cytotoxic T-cell lymphocyte counts and male sex. These data were further supported by the documentation of large between-patient differences in 24-h mean level, peak-trough difference and peak time of plasma cortisol in lung cancer patients [140]. In 161 patients with advanced lung cancer, diurnal salivary cortisol slope was identified as the most critical factor influencing the psychoneurological symptom cluster in multiple linear regression models after adjusting for physical performance status and number of comorbidities [141]. The diurnal slope of salivary cortisol also proved clinically relevant in 202 renal cell cancer patients [138]. Indeed, flatter cortisol slopes were significantly associated with decreased patient survival, a finding similar to that found for this endpoint in lung cancer patients [137].

In contrast, plasma melatonin 24-h patterns appeared quite similar, with a nocturnal peak occurring at 02:00 in each healthy subject, as well as in each early or late-stage lung cancer patient. The relation between the circadian patterns in salivary melatonin and cortisol was further investigated in a case control study involving 40 patients with newly-diagnosed lung cancer and 40 healthy adults [142]. The authors reported that the group of patients had lower salivary melatonin levels and higher salivary cortisol levels with flatter slopes for both hormonal concentrations The multivariate linear regression analysis indicated that the cortisol slope and fatigue score significantly predicted the sleep quality score.

### Relations between immune functions and circadian host or tumor biomarkers

Scarce studies have examined the relationship between host circadian biomarkers and immune functions in patients with lung, kidney, bladder, or esophageal cancer or melanoma. Mazzocoli et al. [140] compared circadian rhythms in circulating Natural Killer (NK), T and B lymphocyte subsets in a group of nine NSCLC patients versus a group of 11 controls. They confirmed the well-known circadian rhythms in circulating immune cell counts in healthy controls, with maxima at daytime for T(CD8), NK-cell subset (CD8dim and CD16+), Vδ2TCR, and at night for T(CD3), T(CD4), and B cells (CD20). In a group of nine stage I–IV NSCLC patients, between-patient differences characterized the 24-h patterns in circulating T(CD3), T(CD8), and B cells. The circadian rhythmicity in circulating NK-cell counts and phagocyte activity were also disrupted in five patients with stage I–II malignant melanoma, compared to 12 healthy controls [143]. The most typical alterations were discoordination between the cytotoxicity rhythms of NK-cells and phagocytes.

The downregulation of clock gene expressions in some human cancers has been associated with altered circadian patterns in the tumor microenvironment, with a possible impact on survival or immunotherapy efficacy. In human malignant melanomas, as well as in human RCCs, the mRNA expression of most clock genes was reduced, whilst displaying disrupted circadian patterns indicative of molecular clock dysfunction, compared to corresponding nonmalignant tissue [144, 145]. Those few melanoma patients whose tumor was characterized with high BMAL1 mRNA expression had prolonged survival, possibly as a result of reduced key DNA-repair enzyme expressions, increased mutational/neoantigen load, and strong intratumoral T-cell infiltration and activation [144]. Moreover, the patients with high BMAL1 tumor expression also achieved significant clinical benefits from immune checkpoint inhibitors [144, 146]. In 11 primary RCC compared to matched healthy tissues, the mRNA expression of clock genes PER2, TIMELESS, and TIPIN was downregulated, whilst that of clock-controlled gene SERPINE1 was upregulated in the tumor cells [147]. In contrast, in the microenvironment of these tumors, the mRNA expression of clock genes BMAL1, REV-ERBα, PER1, PER2, and RORA was upregulated, whilst those of CLOCK, CRY1, and CRY2 declined [148].

The overexpression of PER 2/3, CRY2, and RORα (among other clock genes) in RCC tissues was correlated with longer OS in patients with RCC [149–151]. The survival improvement was associated with the extent of tumor infiltration with T(CD4) and T(CD8) lymphocytes [151], thus highlighting the important role of the interactions between the circadian and the immune systems.

NSCLC patients with high expression of TIMELESS or low expression of RORA, PER1, PER2, or CRY2 had a significantly worse survival prognosis [152]. TIMELESS and RORA are also significantly correlated with immune checkpoint and immune infiltration levels in NSCLC [152]. Clock-related genes were reportedly repressed in human esophageal squamous cell carcinoma samples [153, 154]. In spite of these alterations, PER2 oscillations still occurred in some human esophageal cancer cell lines [154] suggesting that the response to therapy might be enhanced by linking chronotherapy to PER2 expression pattern as a circadian biomarker of interest for personalized chronotherapy.

## Discussion

Here, we discuss how the circadian system can influence the efficacy of ICIs and how the use of circadian biomarkers can help optimize ICI-based treatments in cancer patients.

17 of 18 reported retrospective studies that have examined the influence of ToD administration of ICIs have concluded that predominantly morning or early afternoon infusions significantly improved PFS and/or OS compared with later ToD infusions. These results are consistent with those reported for different vaccines, whose efficacy was usually enhanced following morning (between 9:00 and 11:00) rather than afternoon between 15:00 and 17:00) administration [98, 100]. However, several questions remain unanswered.

When is the optimal circadian rather than daily time of best efficacy and tolerance of ICIs? Despite the consistent finding that early ToD is more effective compared to late ToD, none of the studies reported thus far, have addressed this question. Two main daily cut-off times have been used to differentiate early vs late ToD groups: 12:00–14:00 and 16:00–16:30. Morning dosing might be more advantageous compared to afternoon dosing [32]. Only one-third of the 24-h span has been explored in the 18 studies, thus, questioning whether optimal ICI timing could be located between 18:00 and 9:00. The number of initial ICI infusions to be administered in the morning toward improved efficacy needs to be determined. Thus, four studies highlighted the critical relevance of early ToD administration among the initial four courses. The half-life of ICIs ranges from 14 to 21 days. Thus the circulating steady state is usually reached after 2–4 months [112]. About 8 months are needed to decrease T-cell PD-1 occupancy by 50% after ICIs [113]. The proportion of late ToD courses associated with worse survival also needs to be specified, as it currently ranges from >20 to 67%.

Besides the circadian control of immune functions, other possible explanations have been hypothesized to account for the observed consistency in ICI timing effects.Thus, the ToD of ICI administration could represent a surrogate of socioeconomic status or performance status. Also, imbalances in the total number of ICI administrations between groups of patients treated early or late in the day could moderate treatment efficacy [17]. However, improved efficacy of immunotherapy in randomized trials has been consistently associated with more treatment courses, due to PFS prolongation. Yet, in order to eliminate potential hidden biases, randomized clinical trials are needed. Prospective comparisons should be made between different times of ICI administration to fully appraise the dosing time-related differences in ICI efficacy, which currently appear of the size of a very active new drug.

A possibly overlooked aspect of these studies is the potential impact of sleep, tightly linked to CTS function, on ICI efficacy and optimal ToD administration. Indeed, sleep and immunity are known to be bidirectionally linked, with relevant impact in disease response and health promotion [155]. For instance, in breast cancer patients, aberrant circadian cortisol rhythm was found to be associated with both sleep disruption and suppressed activity of NK cells, ultimately concurring to poorer overall survival [136, 156]. Modern wearable biosensors allow for unintrusive assessment of circadian and sleep cycles, whose alterations have also been associated with immune disruption in night workers [157, 158]. Thus, digital solutions are allegedly going to be paramount in optimization of ICI ToD administration.

Given both the impact of circadian function on outcomes on cytotoxic chemotherapy [62], and the potential for non-pharmacological modulation of ICI efficacy [159, 160], it stands to reason to harness circadian-based behavioural factors to maximize benefits from ICIs.

The available literature suggests that about one-third of the patients with advanced or metastatic cancer display circadian disruption, based upon the suppression or severe dampening of circadian rhythms in rest-activity, cortisol or melatonin secretions or body temperature. These rhythms are critical biomarkers of the circadian coordination system. However, macrophages and other immune cells are endowed with an autonomous molecular clock thus can display rhythmic function and proliferation in vitro, in the absence of endogenous rhythmic signals [161]. Preclinical studies have highlighted the ability of immunologic rhythms to persist despite the complete suppression of the rest-activity and body temperature rhythms [162], whilst altered yet present circadian rhythms in circulating lymphocytes were identified both in tumor-bearing mice with suprachiasmatic nucleus ablation [48], and in cancer patients with suppressed rest-activity or cortisol rhythms [139]. These results highlight the need for specific studies aiming to determine the relations between circadian coordination biomarkers and immunologic rhythms in patients with various malignancies whose response to immunotherapy vary according to dosing time.

Sex-specific differences in the efficacy or tolerability of cancer chronochemotherapy have been reported, as a result of differences in circadian rhythms between male and female patients. An individual patient-level meta-analysis involving data from 345 females and 497 males with metastatic colorectal cancer revealed that males lived significantly longer on chronomodulated oxaliplatin-5-fluouracil-leucovorin compared to conventional chemotherapy. The opposite was observed for women [76]. Experimental and clinical data support an about 6-h delay in the optimal timing of oxaliplatin, 5-fluorouracil and irinotecan, both in mice and in cancer patients [163]. Indeed, many circadian rhythms in hormonal secretions, as well as gene expressions in several tissues display larger amplitudes in females as compared to males, thus highlighting the relevance of sex for circadian drug responses [163, 164]. Regarding chronoimmunotherapy, four of the 18 studies cited here found a trend toward a larger reduction of the relative risk of an earlier death through Early ToD dosing of ICIs in women, as compared to men [11, 12, 21, 29]. Such finding is consistent with larger amplitude rhythms reported in women compared to men [165], although discrepant results have been published on these topics. Also, no ICI timing effects were reported for sex in the 14 other studies. Large meta-analyses have examined whether sex was influential on immunotherapy efficacy irrespective of timing, with two positive and two negative conclusions [166–169]. Thus, prospective studies are needed to establish possible sex-related differences in immunotherapy efficacy, and to link them to circadian and immunologic biomarkers.

Indeed, the main driving mechanisms for ICI chronoefficacy relate to the robust circadian control of immune function, trafficking and ICI responses, that have been shown in experimental models so far. Similar data are expected in cancer patients in order to account for the dosing time-dependent efficacy of ICIs as well as between-patient variations.

The impact of the time of administration for efficacy and tolerability of chemotherapy followed a similar circadian pattern in preclinical models and in cancer patients, resulting in the same timing corresponding to best efficacy and best tolerability [38, 77]. Regarding immunotherapy, an association between the occurrence of adverse events and improved prognosis has been suggested [170, 171]. Consistently with these observations, Karaboué et al. [11] found significantly more skin reactions in patients who received most nivolumab infusions in the morning as compared to afternoon administration. However, morning treatment was both most effective, and associated with significantly less fatigue. Other reports regarding possible time-dependencies in ICIs adverse events are inconclusive [12, 21]. Thus, despite time-dependencies in ICIs-related toxicities deserve careful investigations.

Randomized trials and concomitant translational studies are mandatory for establishing dosing-time dependences of ICI efficacy and tolerability as well as for identifying circadian biomarkers toward the personalization of cancer chronoimmunotherapy. Indeed, it will be important to take into account patients’ robustness and timing or disruption of host circadian rhythms, and sleep disorders, as well as tumor molecular clock functions and genetic mutations.

### Supplementary information

Supplementary Information
