# Supplementary material for: Why does circadian timing of administration matter for immune checkpoint inhibitors’ efficacy?
Source: Br J Cancer. 2024 Jun 4;131(5):783–96. doi: 10.1038/s41416-024-02704-9 (PMC11369086; doi:10.1038/s41416-024-02704-9)
Supplement: Supplementary file 1 — Supplementary Information [file 41416_2024_2704_MOESM1_ESM.pdf]

Karaboué A, Innominato PF, Wreglesworth N, Duchemann B, Adam R, and Lévi F:

Why does circadian timing of administration matter for immune checkpoint inhibitors' efficacy?

## Supplementary Information

**Table S1: Main features of 18 studies reporting effects of time-of-day of administration of immune checkpoint inhibitors**. Main study characteristics are listed in the five left columns. Main impact of ICI timing on PFS and OS are shown in both right columns, with corresponding p-values reported by the authors.

| Studies | Stage IV cancer type | Nb of patients | Therapies | Cut-off time | Median PFS (months) *Before vs After cut-off; p-value* | Median OS (months) *Before vs After cut-off; p-value* |
|---|---|---|---|---|---|---|
| ***Non-small-cell lung cancer*** | | | | | | |
| 1. **Karaboué A et al.** *Cancers (Basel) 2022* | Non-small cell lung cancer | 95 | Nivolumab | 12:54 | 11.3 vs 3.1; p < 0.001 | 34.2 vs 9.6; p < 0.001 |
| 2. **Vilalta A et al.** *Ann Oncol 2021* | Non-small cell lung cancer | 197 | Anti-PD-1 +/- chemotherapy | 12:00 | 6.5 vs 3.2; p=0.066 | 16.1 vs 7.4; p=0.003 |
| 3. **Cortellini A et al.** *J Immunother Cancer 2022* | Non-small cell lung cancer | 180 | Pembrolizumab | 16 :30 | 19.7 vs 6.6 vs; p=0.056 | 47.1 vs 27.8; p=0.11 |
| 4. **Barrios CH et al.** *JCO 2022* | Non-small cell lung cancer | 508 | Nivolumab, Pembrolizumab or Atezolizumab | 16 :00 | Not reported | Not reached for both groups; p=0.5 |
| 5. **Rousseau A et al.** *Eur J Cancer 2023* | Non-small cell lung cancer | 180 | Nivolumab, Pembrolizumab or Atezolizumab | 16:30 | 9.4 vs 4.9; p = 0.020 | 26.2 vs 14.0; p = 0.090 |
| 6. **Karaboué A et al.** *JCO 2023* | Non-small cell lung cancer | 97 | Pembrolizumab +/- chemotherapy | 11:45 | 6.8 vs 1.6; p=0.159 | 42.5 vs 17.0; p=0.010 |
| 7. **Catozzi S et al.** *Eur J Cancer 2024* | Non-small cell lung cancer | 361 | Nivolumab, Atezolizumab, Durvalumab, or Avelumab, +/- chemotherapy | 11:37 | Not reported | 30.3 vs 15.9; p=0.0024 |
| ***Malignant melanoma*** | | | | | | |

| | | | | | | |
|---|---|---|---|---|---|---|
| **8. Qian DC et al.** *Lancet Oncol 2021* | Melanoma | 299 | Nivolumab, Pembrolizumab, or Nivolumab+Ipilimumab | 16 :30 | Not reported | 4.8 y vs not reached, P=0.038 |
| **9. Yeung C et al.** *Immunotherapy 2023* | Melanoma | 121 | Nivolumab, Pembrolizumab, or Nivolumab+Ipilimumab | 13 :00 | 7.6 vs 3.3; p = 0.009 | 24.9 vs 5.5; p < 0.001 |
| **10. Gonçalves L et al.** *Cells 2023* | Melanoma | 73 | Nivolumab, Pembrolizumab, or Nivolumab+Ipilimumab | 14 :00 | 14.9 vs 6.6; p = 0.320 | 38.1 vs 14.9; p < 0.01 |
| ***Urothelial or renal cancers*** | | | | | | |
| **11. Ortego I et al.** *JCO 2022* | Urothelial | 92 | Anti-PD-1 or anti-PD-L1 | 16:30 | 11.38 vs 3.58; p = 0.001 | 14.04 vs 6.80; p=0.001 |
| **12. Dizman N et al.** *JCO 2023* | Renal cell carcinoma | 145 | Nivolumab +/- Ipilimumab | 16:30 | 8.3 vs 4.4; p=0.06 | 46.3 vs 41.7; p=0.20 |
| **13. Fernandez-Mañas L. et al.** *JCO 2023* | Renal cell carcinoma | 56 | ICI | 16 :30 | 10.5 vs 6.3 ; p=0.06 | 56.1 vs 16.9 vs ; p=0.01 |
| **14. Patel J et al.** *JITC 2023* | Renal cell carcinoma | 201 | Pembrolizumab, Nivolumab, or Nivolumab+Ipilimumab | 13 :00 | Not reported | 58 vs 34 ; P=0.017 |
| ***Gastrointestinal cancers*** | | | | | | |
| **15. Nomura M et al.** *Esophagus 2023* | Squamous cell carcinoma of esophagus | 62 | Nivolumab | 13:00 | 5.5 vs 2.5; p=0.002 | 19 vs 12; p=0.036 |
| **16. Ishizuka Y et al.** *JCO 2024* | Gastric cancer | 248 | Nivolumab | 14 :00 | 2.3 vs 1.6 ; p < 0.001 | 7.6 vs 3.9 ; p < 0.001 |
| **17. Pascale A et al.** *JCO 2024* | Hepatocellular carcinoma | 131 | Atezolizumab +/- Bevacizumab | 13:00 | Not reported | 18.7 vs 11.5; p=0.015 |
| ***Pan cancer*** | | | | | | |
| **18. Rensburg, HJJv et al.** *JCO 2022* | Head and neck, breast, ovarian, melanoma, and other solid tumours | 106 | Pembrolizumab | 12 :00<br>15 :11<br>16 :30 | No difference | No difference |
